# Apple skin patterning is associated with differential expression of *MYB10*

**DOI:** 10.1186/1471-2229-11-93

**Published:** 2011-05-20

**Authors:** Adriana Telias, Kui Lin-Wang, David E Stevenson, Janine M Cooney, Roger P Hellens, Andrew C Allan, Emily E Hoover, James M Bradeen

**Affiliations:** 1 Plant Science and Landscape Architecture Department, University of Maryland 2102 Plant Sciences Building, College Park, MD 21201, USA; 2 Plant and Food Research, Mt Albert Research Centre Private Bag 92169, Auckland, New Zealand; 3 Plant and Food Research, Ruakura Research Centre Private Bag 3123, Hamilton, New Zealand; 4 School of Biological Sciences, University of Auckland, Private Bag 92019, Auckland, New Zealand; 5 Department of Horticultural Science, University of Minnesota 305 Alderman Hall, 1970 Folwell Ave., St. Paul, MN 55108, USA; 6 Department of Plant Pathology, University of Minnesota 495 Borlaug, 1991 Upper Buford Cir., St. Paul, MN 55108, USA

## Abstract

**Background:**

Some apple (*Malus *× *domestica *Borkh.) varieties have attractive striping patterns, a quality attribute that is important for determining apple fruit market acceptance. Most apple cultivars (e.g. 'Royal Gala') produce fruit with a defined fruit pigment pattern, but in the case of 'Honeycrisp' apple, trees can produce fruits of two different kinds: striped and blushed. The causes of this phenomenon are unknown.

**Results:**

Here we show that striped areas of 'Honeycrisp' and 'Royal Gala' are due to sectorial increases in anthocyanin concentration. Transcript levels of the major biosynthetic genes and *MYB10*, a transcription factor that upregulates apple anthocyanin production, correlated with increased anthocyanin concentration in stripes. However, nucleotide changes in the promoter and coding sequence of *MYB10 *do not correlate with skin pattern in 'Honeycrisp' and other cultivars differing in peel pigmentation patterns. A survey of methylation levels throughout the coding region of *MYB10 *and a 2.5 Kb region 5' of the ATG translation start site indicated that an area 900 bp long, starting 1400 bp upstream of the translation start site, is highly methylated. Cytosine methylation was present in all three contexts, with higher methylation levels observed for CHH and CHG (where H is A, C or T) than for CG. Comparisons of methylation levels of the *MYB10 *promoter in 'Honeycrisp' red and green stripes indicated that they correlate with peel phenotypes, with an enrichment of methylation observed in green stripes.

**Conclusions:**

Differences in anthocyanin levels between red and green stripes can be explained by differential transcript accumulation of *MYB10*. Different levels of *MYB10 *transcript in red versus green stripes are inversely associated with methylation levels in the promoter region. Although observed methylation differences are modest, trends are consistent across years and differences are statistically significant. Methylation may be associated with the presence of a TRIM retrotransposon within the promoter region, but the presence of the TRIM element alone cannot explain the phenotypic variability observed in 'Honeycrisp'. We suggest that methylation in the *MYB10 *promoter is more variable in 'Honeycrisp' than in 'Royal Gala', leading to more variable color patterns in the peel of this cultivar.

## Background

Apple peel color is one of the most important factors determining apple market acceptance. In general, red cultivars are the most preferred, and within a cultivar more highly colored fruits are favored [1]. Consumer preferences vary from country to country and region to region: New Zealand consumers prefer striped apples, consumers in Nova Scotia, Canada prefer blushed apples, while consumers in British Columbia, Canada are more accepting of a range of apple types [2]. Peel pigments not only affect visual appeal, they also contribute to the fruit's nutritional value. Apples have been associated with lowered risks of cancer and cardiovascular diseases, which are thought to be caused by oxidative processes. Polyphenolics, including anthocyanins which are the red pigments in apple peels, have been found to be the major source of antioxidants in apple [3]. Antioxidants are mainly localized in the apple peel, but cultivars exhibit a wide variation in the distribution pattern [4,5]. Anthocyanin accumulation in apple peels can be affected by genetic, environmental, nutritional and cultural factors, the stage of maturity of the fruit, and by the microenvironment within the canopy [6,7].

**Figure 1:**
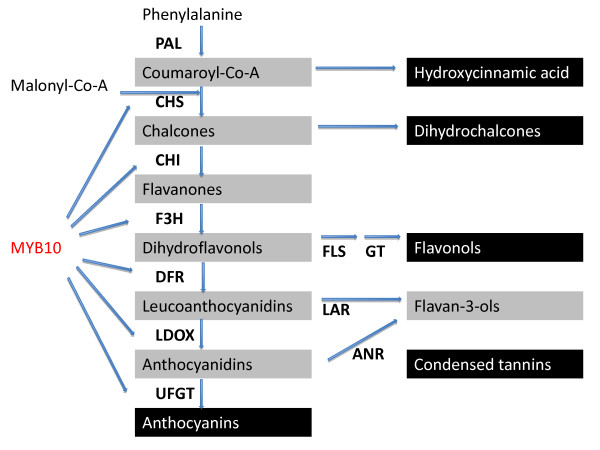
**Schematic representation of the flavonoid biosynthetic pathway in apple regulated by *MYB10***. Flavonoid intermediates (gray boxes) and end products (black boxes) are indicated. Enzymes required for each step are shown in bold uppercase letters (PAL, phenylalanine ammonia lyase; CHS, chalcone synthase; CHI, chalcone isomerase; F3H, flavanone-3β-hydroxylase; FLS, flavonol synthase; GT, unidentified enzyme encoding a glycosyl transferase for flavonol glycone synthesis; DFR, dihydroflavonol-4-reductase (denoted as DFR1 in the text); LAR, leucoanthocyanidin reductase; LDOX, leucoanthocyanidin dioxygenase; ANR, anthocyanidin reductase; UFGT, UDP-glycose:flavonoid-3-O-glycosyltransferase (adapted from [17]).

The main anthocyanin identified in apple skin is cyanidin 3-galactoside, while cyanidin 3-glucoside levels are very low [8-10]. Two categories of genes affect the biosynthesis of anthocyanin. The first category encodes enzymes required for pigment biosynthesis (structural or biosynthetic genes), which have been widely studied in apple [8-11] (Figure 1). The second category is comprised of transcription factors, which are regulatory genes that influence the intensity and pattern of anthocyanin accumulation and control transcription of different biosynthetic genes. At least three families, MYB, bHLH and WDR, have been found to be involved in the regulation of anthocyanin synthesis, but the specific classes and genes involved vary depending on the species [12-14].

In apple, three research groups have independently identified an R2R3 MYB transcription factor responsible for anthocyanin accumulation in fruit. The loci have been named *MYB1*, *MYB10 *and *MYBA *[12,15-17]. The coding region of *MYBA *is 100 and 98% identical to *MYB1 *and *MYB10*, respectively [15]. In addition, *MYB10 *and *MYBA *have been mapped to the same region on linkage group 9 [15,18]. Subsequent experiments have shown that *MYB1*, *MYB10 *and *MYBA *are likely to be allelic [19] and more-over, at this locus in the current apple genome assembly, there is only one MYB present [20]. Based on this evidence, in this research article, we consider *MYB10 *to exist as a single locus with *MYBA *and *MYB1 *representing alleles of the *MYB10 *locus.

Transcript levels of the *MYB1 *allele correlate with anthocyanin accumulation and are higher in red fruit peel sectors (more exposed to light) and in red peel cultivars than in green peel sectors or cultivars [17]. Transcript levels of *MYB1 *increased in dark-grown apples once exposed to light, providing additional evidence of its role as an anthocyanin regulator. *MYB1-1*, a sequence variant of the *MYB1 *allele, co-segregates with red skin color [17,21]. Transcription at the *MYB10 *locus strongly correlates with peel anthocyanin levels and this gene is able to induce anthocyanin accumulation in heterologous and homologous systems [12]. In addition, *MYB10 *co-segregates with the *Rni *locus, a major genetic determinant of red foliage and red color in the core of apple fruit [18]. Consistently, the expression of several anthocyanin pathway genes was found to be regulated by MYB10 and MYB1 [12,17] (Figure 1). In apple, two candidate bHLH transcription cofactors (bHLH3 and bHLH33) are also needed for activating promoters of anthocyanin structural genes and *MYB10 *[12,22].

Repressors of anthocyanin production were also identified within the MYB class of transcription factors, including *MdMYB17 *in apple [23], *FaMYB1 *in strawberry [24] and *AtMYBL2 *in Arabidopsis [25,26]. *FaMYB1 *is up-regulated jointly with late anthocyanin pathway genes [24]. Expression of *AtMYBL2 *is also coordinately up-regulated by the MYB-bHLH-WDR activation complex [26,27]. In Arabidopsis a transcriptional regulatory loop has been postulated whereby AtPAP1 (MYB) is a positive regulator of AtTT8 (bHLH) [28], and AtTT8 is an activator of *AtMYBL2 *expression [26] which then negatively regulates the expression of *AtTT8*. It is suggested that the repressors' role is to balance anthocyanin levels produced at later stages of color response.

**Figure 2:**
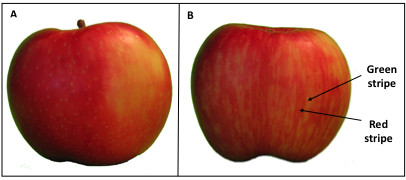
**Different types of fruit peel pigment patterns in 'Honeycrisp' apple**. Distribution of anthocyanin in apple peels of blushed A) and striped B) fruits of 'Honeycrisp', indicating regions classified as red or green stripes.

'Honeycrisp', an increasingly important apple cultivar developed at the University of Minnesota, produces fruits that can adopt two basic peel color patterns: blushed or striped (Figure 2). For the purposes of this study, fruits are defined as striped when the color alternates between vertically elongated regions in some or all portions of the peel. Fruits are termed blushed when the surface is partly covered with a red tinge that is not broken. These two phenotypic categories are mutually exclusive. In 'Honeycrisp' both kinds of fruit may be present on the same tree, a characteristic that has not been described in other cultivars. The molecular basis of this phenomenon is unknown.

Different mechanisms can cause variegation in plants, including chimeras [29], transposable element activity [30] and cytosine methylation [31]. Previous results do not provide evidence for a chimeral source of variegation in the case of 'Honeycrisp', since the phenotype is not stable after propagation [32] as would be expected if changes were caused by a periclinal chimera. Microscopic observations indicated that the difference between stripes is due to a reduction in pigment accumulation in the paler stripes, both in the epidermis and in the first hypodermal layers [32].

Activation and suppression of transposable elements may be responsible for some of the genetic variation that occurs in peel color in pome fruits [33]. Transposable elements have been identified in apple [34-41] but to date there is no evidence associating transposable elements with fruit peel variegation. The presence of transposable elements can affect gene expression both at the transcriptional (e.g. through the introduction of an alternative transcription start site), and at the post-transcriptional level [42].

Cocciolone and Cone [31] reported that striped patterns of anthocyanin accumulation in maize were due to differential DNA methylation in the 3' untranslated region of *Pl-Bh*, a MYB transcription factor regulating anthocyanin accumulation. Methylation was found to be inversely correlated with *Pl-Bh *mRNA levels in variegated plant tissues. The authors hypothesized that early during development, the *Pl-Bh *gene would be differentially methylated and this methylation would be more or less maintained through subsequent cell divisions, producing clonal sectors in plant tissues of predominantly pigmented cells (unmethylated) and sectors of predominantly unpigmented cells (methylated). Sekhon and Chopra [43] identified a gene called *Ufo1 *that controls methylation levels in *p1*, a gene that regulates phlobaphene biosynthesis in maize, and whose activity may also produce variegation in the maize pericarp. Ectopic expression of *P1-wr *correlated with hypomethylation of an enhancer region, 5 Kb upstream of the transcription start site. It is not known whether methylation is responsible for color differences in apple.

We therefore sought to understand the molecular mechanism responsible for 'Honeycrisp' color pattern regulation and instability. We also included in this study two stably striped cultivars ('Royal Gala' and 'Fireside'), a stably blushed cultivar ('Connell Red', a sport of 'Fireside') and other cultivars differing in the degree of peel pigmentation. Our results showed that variation in pigment accumulation between red and green stripes correlates with anthocyanin levels, and the steady state mRNA levels of both the anthocyanin biosynthetic genes and the transcription factor *MYB10*. Sequence variation in the *MYB10 *region upstream of the translation start site (referred to as "promoter" for simplification) and coding region does not explain the observed phenotypes. The promoter and coding regions of *MYB10 *were examined in red and green stripes for DNA methylation levels and a 900 bp region, starting 1400 bp upstream of the predicted translation start site, was found to be highly methylated in both 'Honeycrisp' and 'Royal Gala'. Red stripes were associated with lower methylation across the promoter of *MYB10 *in 'Honeycrisp' and to a lesser degree in 'Royal Gala', but no differences were found between blushed 'Honeycrisp' green and red peel regions.

## Results

### Red stripes have higher anthocyanin accumulation and transcript levels of biosynthetic genes

**Figure 3:**
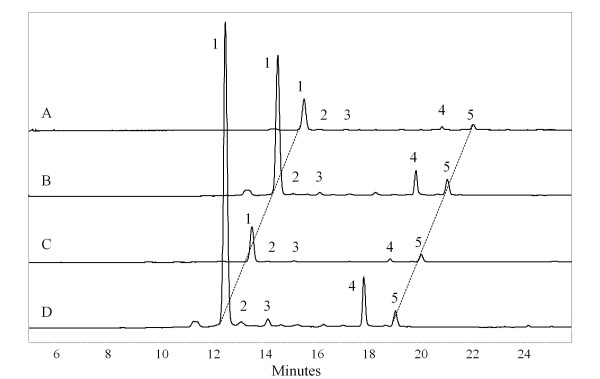
**The levels of cyanidin-3-galactoside differ in red and green stripes of 'Honeycrisp' and 'Royal Gala'**. HPLC traces at 520 nm of A) green and B) red stripes of 'Honeycrisp' and C) green and D) red stripes of 'Royal Gala'. Peak identification (observed molecular ion/major fragment, masses in Da): 1 - Cyanidin-3-galactoside (M^+ ^= 449, 287); 2 - Cyanidin-3-glucoside (M^+ ^= 449, 287); 3 - Cyanidin pentoside (M^+ ^= 419, 287 most likely the arabinoside); 4 and 5 - Tentatively identified (ions were low intensity) as pelargonidin derivatives (M^+ ^= 557, 395, 271 Da, implies presence of pelargonidin, hexoside sugar and an unidentified species; mass 124). Chromatograms are offset on the time axis by one minute for clarity.

Red stripes of 'Royal Gala' and 'Honeycrisp' contained approximately eight and four times as much anthocyanin as green stripes (83 vs. 10 and 38 vs. 10 μg/g of anthocyanin monoglycoside equivalent for 'Royal Gala' and 'Honeycrisp', respectively). In all cases, the major anthocyanin detected was cyanidin-3-galactoside (Figure 3).

**Figure 4:**
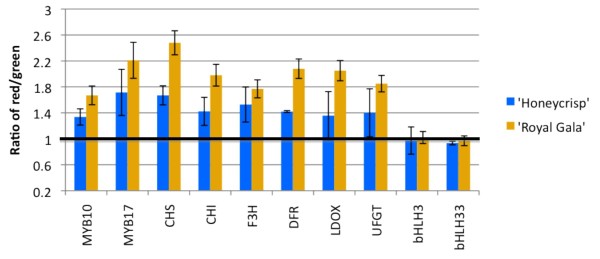
**Transcript levels of apple anthocyanin genes determined by real-time PCR**. Values indicate the ratio between the normalized transcript levels (relative to *actin*) of structural genes (*CHS*, chalcone synthase; *CHI*, chalcone isomerase; *F3H*, flavanone-3β-hydroxylase; *DFR*, dihydroflavonol-4-reductase (denoted as *DFR1 *in the text); *LDOX*, leucoanthocyanidin dioxygenase; *UFGT*, UDP-glycose:flavonoid-3-O-glycosyltransferase) and transcription factors (*MYB10*, *MYB17*, *bHLH3 *and *bHLH33*) in red and green stripes of 'Honeycrisp' and 'Royal Gala' as indicated. Reactions were performed in triplicate. Error bars are SE.

We subsequently compared the transcript levels of regulatory genes *MYB10*, *MYB17*, *bHLH3 *and *bHLH33 *and biosynthetic genes *CHS, CHI, F3H, DFR1, LDOX, UFGT*, in RNA isolated from red and green stripes of 'Royal Gala' and 'Honeycrisp' (Figure 4). *MYB10 *and *MYB17 *transcript levels correlated with anthocyanin concentration in both 'Honeycrisp' and 'Royal Gala', with higher mRNA levels in red stripes as compared to green stripes (ratios significantly larger than 1, p ≤ 0.05). Transcript levels of structural genes followed the same pattern as those of *MYB10 *and *MYB17*. Levels of the two bHLH transcription factors did not differ in green and red stripes (p ≤ 0.05), and therefore correlated poorly with anthocyanin concentration. These results reveal differential transcript accumulation of *MYB10 *and *MYB17 *in differentially pigmented stripes, which in turn results in a corresponding modulation of transcript levels of structural genes. *MYB10 *is a known activator of the apple anthocyanin pathway [17] and *MYB17 *has been shown to inhibit steps in the anthocyanin pathway [23] and has high sequence similarity to *AtMYB4*, a repressor of the phenylpropanoid pathway [44,45]. We decided to further characterize *MYB10 *coding and upstream regions in order to determine whether sequence polymorphisms can explain different pigmentation patterns.

### Low sequence diversity in the *MYB10 *coding region in 'Honeycrisp', 'Connell Red' and 'Fireside'

To study the possibility that sequence differences are the cause of differential color patterns in the peel, we sequenced a total of 94 cDNA clones of the 'Honeycrisp' *MYB10 *coding region: 47 from a phenotypically uncharacterized 'Honeycrisp' fruit (harvested in late August when pigment pattern could not yet be conclusively determined), 24 from a mature striped and 23 from a mature blushed fruit. Ninety-two percent of the sequences obtained were 100% identical to *MYB1-1*, an allele of the *MYB10 *locus [17]. We found three single nucleotide polymorphisms (SNP) that produce changes in protein sequence, but since each one appeared only once in our dataset, and in phenotypically different apples, they most likely represent amplification or sequencing errors. These results indicate low levels of sequence diversity in the *MYB10 *coding region in 'Honeycrisp', with no evidence suggesting that the blushed/striped phenomenon is associated with modifications at the primary DNA sequence level within the coding region. *MYB10 *coding sequences from the striped cultivar Fireside (24 clones) and 'Connell Red' (23 clones), a stably blushed sport of 'Fireside', are identical to that of the most abundant version found in 'Honeycrisp' and the previously published *MYB1-1 *sequence--supporting our conclusion that differences in primary DNA sequence are not the source of differential patterns of apple peel pigment accumulation.

### No size variation in *MYB10 *promoter region among apple cultivars

We amplified three fragments (-2029 to -1229, -1411 to -678, and -677 to 47; nucleotide positions on the Genbank accession EU518249 relative to translation start site) collectively spanning about 2 Kb of the *MYB10 *promoter. PCR results did not indicate any fragment size differences among blushed and striped 'Honeycrisp', 'Connell Red' and 'Fireside' DNA, suggesting no large insertion or deletions were present. We sequenced the PCR products of each of these fragments from three independent reactions and found no sequence differences between blushed and striped 'Honeycrisp', or between 'Connell Red' and 'Fireside', although there were 14 SNPs between 'Honeycrisp' and the other two cultivars.

### Neither presence nor transcription of a TRIM element explains apple peel phenotypic variation

**Figure 5:**
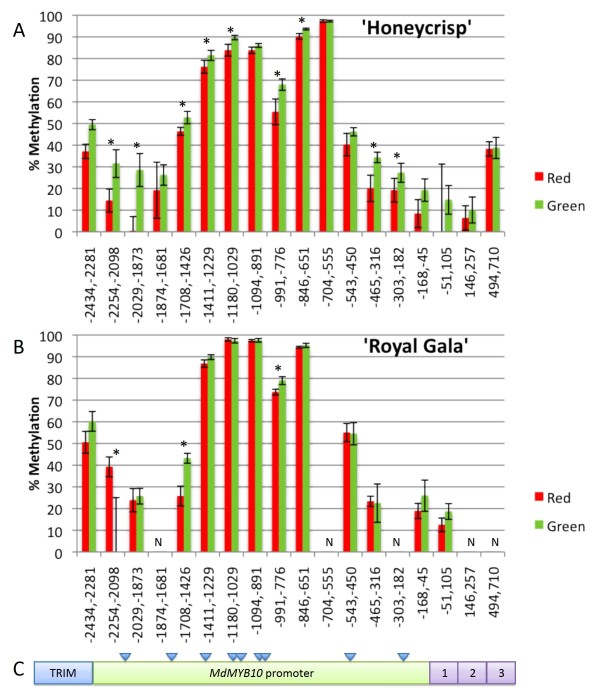
**Methylation levels across *MYB10 *in 'Honeycrisp' and 'Royal Gala'**. Percent methylation in A) 'Honeycrisp' and B) 'Royal Gala' green and red stripes across the *MYB10 *locus (Genbank accession EU518249) (C), estimated using an assay combining McrBC digestions and real-time PCR amplification. Percent methylation indicates the proportion of copies cut by McrBC. Values on the X-axis indicate the location of the primers used relative to the ATG translation start site of *MYB10*. Panel C indicates the relative location of the TRIM element, the *MYB10 *promoter and three exons (1, 2, 3); this figure is not to scale. The blue triangles indicate the approximate positions of E-box motifs within the promoter region. The calculated % methylation for the -51 to 105 fragment in 'Honeycrisp' and the -2254 to -2098 in 'Royal Gala' were negative, therefore a value of 0 is indicated in the plot. Methylation in the -704 to -555 fragment in 'Royal Gala' could not be estimated given the extremely low template levels in the McrBC treated sample. The -1874 to -1681, -303 to -182, 146 to 257 and 494 to 710 fragments were not evaluated in 'Royal Gala' (N). Reactions were performed in triplicate and two or three independent digestions were used. Error bars are SE and stars indicate significant differences (p ≤ 0.05).

Within the Plant & Food *Malus *gene database [46] was a DNA sequence identical to Genbank accession EU518249, the promoter of *MYB10*. Further upstream from this sequence, between positions -3038 and -2420 from the ATG translation start site of *MYB10 *(EU518249, 'Royal Gala') was a sequence with 85% identity to a *Malus *TRIM element (AY603367), a terminal-repeat retrotransposon in miniature [34]. We checked for the presence of a TRIM element upstream of the *MYB10 *locus in 'Honeycrisp' (blushed and striped), 'Connell Red', 'Fireside', '1807' (green selection) and 'Geneva' (ultra red cultivar) via PCR, combining a primer designed from the TRIM element (TRIM forward primer) with one designed from the promoter region of *MYB10 *(primer -1873). Results confirmed the presence of the TRIM element in each of these cultivars in a position identical to that observed in 'Royal Gala' (Figure 5C). We subsequently cloned and sequenced three PCR products from 'Honeycrisp' (blushed and striped), 'Connell Red' and 'Fireside'. Half of the fragments yielded sequences showing 99% or more identity to the previously published (EU518249) upstream region of 'Royal Gala' *MYB10*. The other sequences were probably amplifications from insertions of similar TRIM elements located elsewhere in the genome, with percent identities to TRIM ranging from 40 to 56.5%.

We tested for TRIM transcript presence in blushed and striped 'Honeycrisp', 'Connell Red', 'Fireside', 'Geneva' (ultra red cultivar) and 'Honeygold' (green cultivar), and found it to be transcribed in all cases. However, a fragment spanning a portion of the TRIM element and extending 500 bp into the upstream region of *MYB10 *did not amplify from total RNA, indicating that transcription from the TRIM element did not extend into *MYB10 *in these cultivars. Overall, results indicated that neither the presence of the TRIM element in the *MYB10 *promoter region nor its transcription explained the differences in peel pigment accumulation among the cultivars studied.

### Increased methylation levels in green stripes

**Figure 6:**
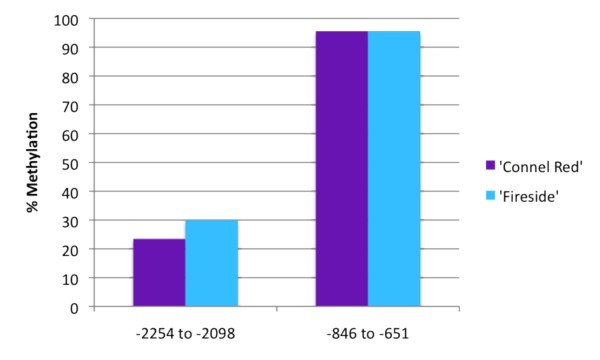
**Methylation levels in two *MYB10 *promoter regions in 'Fireside' and 'Connel Red'**. Percent methylation in a low (-2254 to -2098) and a high (-846 to -651) methylation region of the *MYB10 *promoter (GenBank accession EU518249) in 'Connel Red' and 'Fireside' peel DNA (2007 fruit samples). Percent methylation was calculated using an assay combining McrBC digestions and real-time PCR and indicates the proportion of copies cut by McrBC. The X-axis indicates nucleotide positions relative to the ATG translation start site. Reactions were performed in triplicate and two independent digestions were used.

DNA samples from green and red stripes of 'Honeycrisp' (2007 samples) and 'Royal Gala' were treated with the methylation-sensitive endonuclease McrBC to ascertain whether the observed differences in transcript accumulation were associated with methylation differences in the promoter or coding region of *MYB10 *(Figure 5). McrBC preferentially cuts DNA containing methylcytosine on one or both strands, between two recognition sites [5'...Pu^m^C(N_40-3000_)Pu^m^C...3']. McrBC treated and mock-digested templates were compared using real-time PCR, and percent methylation was calculated. In total, 18 fragments starting at the transposon insertion and spanning 2.5 Kb of the promoter region and three exons of *MYB10*, were evaluated. Results indicated that a region of the *MYB10 *promoter, encompassing the fragments between nucleotide positions -1411 and -555 is highly methylated (above 60%) in both cultivars. 'Connell Red' and 'Fireside' had low methylation (20-40%) in the -2254 to -2098 fragment and high methylation (95%) in the -846 to -651 fragment, indicating a similar pattern of *MYB10 *methylation in these cultivars relative to those observed in 'Royal Gala' and 'Honeycrisp' (Figure 6).

**Figure 7:**
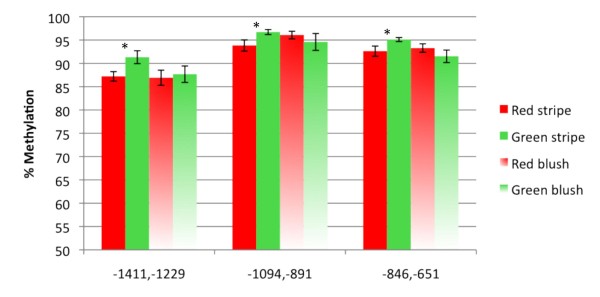
**Methylation levels in three *MYB10 *promoter regions in striped and blushed 'Honeycrisp' peels**. Comparison of percent methylation in the highly methylated region (-1411 to -651) of the *MYB10 *promoter (GenBank accession EU518249) between red and green stripes, and red and green areas of blushed 'Honeycrisp' (2008 fruit samples). Percent methylation was calculated using an assay combining McrBC digestions and real-time PCR and indicates the proportion of copies cut by McrBC. The X-axis indicates nucleotide positions relative to the ATG translation start site. Reactions were performed in triplicate and two independent digestions were used. Error bars are SE and stars indicate significant differences (p ≤ 0.05).

Green stripes of 'Honeycrisp' (2007 samples) showed higher overall methylation levels than red stripes throughout the promoter region (Figure 5A). The -704 to -555 fragment was omitted from this comparison since quantification in the McrBC digested samples was highly variable due to extremely low template levels, indicating that this region was so highly methylated that treatment with McrBC resulted in nearly complete digestion of the template DNA. Sequence analysis indicated that differences in predicted methylation levels between regions were not due to difference in the number of potential McrBC recognition sites (data not presented). Similar results were obtained for 2008 fruits, but overall methylation levels were higher than in 2007 and differences between red and green stripes were even greater (Figure 7). These results indicate that while methylation levels are variable between years, green stripes are consistently associated with higher methylation of *MYB10 *promoter regions. Similar trends were observed in 'Royal Gala' for some of the fragments, except that the differences between red and green stripes were smaller. In total, higher methylation levels were observed for 'Royal Gala' than 'Honeycrisp' (Figure 5B).

In contrast to 'Honeycrisp' red and green stripes, we hypothesized that color differences between red (exposed to light) and green (unexposed to light) regions of the peel of blushed apples are only due to light effects and not to differences in methylation levels. We therefore compared methylation percentages in red (exposed) and green (unexposed) areas of blushed apples and red and green stripes. Results indicated no significant differences (p ≤ 0.05) between red and green regions of the peel of blushed apples. Interestingly, in two out of the three regions studied (-1411 to -1229 and -846 to -651), red stripes have methylation levels comparable to those in the exposed peel portions of blushed apples, while green stripes have methylation levels higher than those of red stripes or red and green regions of blushed apples (Figure 7).

**Figure 8:**
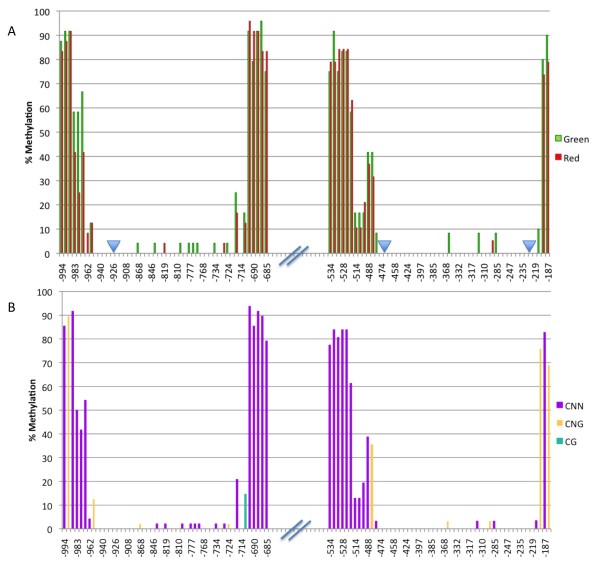
**Methylation levels in 'Honeycrisp' evaluated using bisulfite sequencing**. Comparison of percent methylation in two regions (-1007 to -684 and -534 to -184) of the *MYB10 *promoter (GenBank accession EU518249) between red and green stripes (A) and among three methylation contexts (B). Percent methylation was calculated based on the cytosine methylation status of a number of clones after bisulfite conversion and sequencing. The X-axis indicates nucleotide positions relative to the ATG translation start site. E-box motifs are indicated with blue triangles in panel A. Values in panel B represent the average of green and red stripes.

Bisulfite sequencing offers greater resolution than McrBC-based methods for the detection of methylated cytosines. Building on McrBC results, we next pursued bisulfite sequencing of *MYB10 *promoter regions from 'Honeycrisp' and 'Royal Gala'. Preliminary bisulfite sequencing experiments indicated that cytosine methylation in the promoter region of *MYB10 *is found in all three methylation contexts (i.e. CHH, CHG, and CG, where H is A, C or T). To avoid amplification bias, we therefore designed degenerate PCR primers to target two different promoter regions. This severely constrained areas that could be targeted, and amplification upstream of -1007 was ultimately unsuccessful using unbiased primers. A comparison of methylation levels between red and green stripes in the -1007 to -684 and -534 to -184 regions confirmed that green stripes are more highly methylated than red stripes (9.3 and 5.2% difference respectively), with 80% and 65% of cytosines showing higher methylation levels in green than in red stripes in the -1007 to -684 and -534 to -184 regions respectively (Figure 8A). Further analysis of the -1007 to -684 region indicated that clones obtained from green stripes have higher overall methylation levels than those obtained from red stripes (Additional files 1 and 2). Observed methylation differences between red and green stripes are modest, but actual differences may be greater. Although great care was taken, manual isolation of red and green stripes from 'Honeycrisp' peels was imprecise, resulting in tissue samples that were substantially enriched for red or green stripes but not entirely devoid of contaminating tissues. Thus, DNA samples used for McrBC- and bisulfite-based analyses, while predominantly derived from the target tissue (red or green stripes) likely represent a mixture of DNA, partially obscuring methylation differences between sources.

Consistent with our preliminary results, different cytosine contexts did not exhibit distinct methylation patterns; all cytosine contexts showed high methylation levels in highly methylated regions and vice versa (Figure 8B). Overall, CHH and CHG methylation was highest (20.2 and 16.9% respectively) and CG methylation was lowest (1.6%). A sequence alignment for the -1007 to -684 region is presented in Additional file 2.

## Discussion

### Anthocyanin genes transcript levels correlate with striped patterns

Anthocyanin and transcript quantifications performed in this study suggested a possible mechanism controlling pigment patterns in apple fruit peels. We have found that green stripes are associated with lower anthocyanin accumulation, which is explained by reduced transcript levels of all the anthocyanin pathway genes evaluated, including the structural genes in the pathway, and *MYB10*, a transcription factor which regulates them. An additional gene evaluated in this study, *MYB17*, an apple transcription factor that represses anthocyanin synthesis [23] was transcribed in a similar manner to *MYB10*. As *MYB17 *is not elevated in green sectors relative to red, we considered *MYB10*, the main transcription factor regulating the pathway in apple [12,15,17,21], to be the most likely candidate to be involved in peel variegation. We therefore sought to identify a mechanism responsible for *MYB10 *transcript level differences.

### Variegation in apple peels is associated with *MYB10 *methylation mosaicism

Our results indicate an inverse association between methylation levels in the *MYB10 *promoter and anthocyanin accumulation in striped apple peels. As previously suggested by Cocciolone and Cone [31] for maize, we hypothesized that early in apple fruit development, differences in *MYB10 *methylation are present among individual cells. Throughout fruit growth, these differentially methylated cells give rise to sectors of peel varying in their ability to accumulate anthocyanins. Our results indicate that DNA methylation in the promoter of *MYB10 *is associated with reduced transcript accumulation. Specifically, we propose that differential methylation of *MYB10 *promoter regions in red *vs. *green stripes of the 'Honeycrisp' peel results in differential accumulation of the *MYB10 *transcript, which in turn determines both transcription of anthocyanin structural genes and pigment accumulation. DNA methylation may affect *MYB10 *transcription through interference with the RNA-polymerase transcription complex or by preventing binding of additional factors required for transcription. In Arabidopsis, genome wide studies of DNA methylation have found that methylation within regulatory regions is rare and probably selected against, as it may interfere with transcription initiation [47]. Our results suggest that high levels of methylation in certain promoter regions of a key transcription factor in the flavonoid biosynthetic pathway in apple may play a regulatory role, but it is not inhibitory for gene activity. It is possible that since apple trees are clonally propagated and stay in production for many years (i.e. fruit peel tissue does not derive from cells that have undergone recent meiosis), mechanisms of epigenetic regulation might not be identical to what has been described in more widely studied species such as Arabidopsis and rice.

Different methylation levels early in apple fruit development could be mitotically maintained from those in the meristematic cells that gave origin to the fruit, or could result from demethylation or *de novo *methylation. Previous results in 'Honeycrisp' suggest that there is at least some mitotic maintenance of methylation states, given that trees clonally propagated from buds on branches with exclusively blushed fruits, tend to produce a higher percentage of blushed fruit [32]. Nonetheless, results from the same study indicated that additional factors can influence the pattern in the peel, namely position of the fruit on the tree and crop load.

The presence of a TRIM transposable element in an upstream region of the *MYB10 *promoter might influence the changes in methylation observed between different regions of the peel but neither its presence *per se *nor its transcription appears to be responsible for determining peel pigmentation. The TRIM element identified in this study is located 2.5 Kb upstream of the predicted translation start site, and is present in 'Honeycrisp', 'Royal Gala', and five other cultivars with peel pigmentation ranging from green to ultra red. Lippman et al. [48] indicated that in Arabidopsis transposable elements can determine epigenetic gene silencing when inserted within or very near (<500 bp) a gene. The effect of a transposable element 2.5 Kb upstream of the coding region is unknown. We did not find any evidence of transposable element sequences within the highly methylated promoter region of *MYB10.*

Within the most methylated region of the *MYB10 *promoter in this study (-1411 to -555; Figure 5) are five putative E-box motifs [22] which are bHLH-related cis-acting elements (CACATG) [49,50]. Methylation was absent at the three E-box motifs evaluated using bisulfite sequencing, but highly methylated areas occurred a few nucleotides upstream or downstream of these motifs. This may suggest a potential regulatory role for one or more of these motifs. An assessment of the presence of other types of epigenetic marks such as histone methylation can shed additional light on the mechanism involved in *MYB10 *regulation. Our results show that methylation followed the same pattern in all three cytosine methylation contexts, with alternation of high and low methylation regions. The high methylation levels observed for CHH and CHG sites, and low methylation levels for the CG context, indicate a pattern not representative of what is generally observed in flowering plants [51,52]. Broader survey of methylation patterns throughout the apple genome is warranted.

The unstable nature of pigment patterning in 'Honeycrisp' could be a result of a more variable cell to cell methylation pattern than is present in other cultivars producing fruit with consistent pigment patterns, such as 'Royal Gala', 'Fireside' and 'Connell Red'. We speculate that the occurrence of stripes in 'Honeycrisp' is a consequence of higher than normal methylation levels in the *MYB10 *promoter region in the green stripes, something that occurs only in some fruit and to varying degrees. In contrast, *MYB10 *methylation levels and thus peel pigmentation remains more constant in 'Royal Gala'.

## Conclusions

Differences in anthocyanin levels between red and green stripes can be explained by differential transcript accumulation of *MYB10*, a transcription factor that regulates the anthocyanin pathway in apple. Different transcript levels of *MYB10 *in red versus green stripes are inversely associated with methylation levels in its promoter, especially along a 900 bp region upstream of the translation start site. Although observed methylation differences are modest, trends are consistent across years and differences are statistically significant. Methylation might be associated with the presence of a TRIM element within the promoter region, but the presence of the TRIM element alone cannot explain the phenotypic variability observed in 'Honeycrisp'. We suggest that methylation in the *MYB10 *promoter is more variable in the phenotypically plastic 'Honeycrisp' than in the more consistently striped 'Royal Gala'. Differential methylation of the 'Honeycrisp' *MYB10 *promoter alters accumulation of the *MYB10 *transcript, in turn altering both transcription of anthocyanin structural genes and pigment accumulation.

## Materials and methods

### Plant material

Leaf samples of 'Honeycrisp', 'Connell Red', 'Fireside', '1807', 'Honeygold' and 'Geneva' apple were collected in early spring of 2005 and fruits of the same cultivars were collected at maturity during the 2005, 2006, 2007 and 2008 growing seasons from trees at the Horticultural Research Center in Chanhassen, Minnesota. In February 2008 ('Royal Gala') and 2010 ('Honeycrisp') fruits were harvested at Plant and Food Research orchards (Nelson, New Zealand). 'Royal Gala' apples grown in Chile were purchased in a Minnesota grocery store in April 2008 to be used for methylation experiments. For the *MYB10 *characterization experiments, whole fruit peels were used. For anthocyanin quantification, transcript analyses and methylation studies, red and green stripes were carefully separated using a razor blade. Since stripes cannot be absolutely classified as green or red, samples are more accurately described as "red stripe enriched" or "green stripe enriched". Both green and red stripes were obtained from the same region of the peel at each time, preventing the possibility that the "red stripe enriched" sample would also be enriched for fruit peel regions more exposed to light and vice versa. For comparisons between different blushed fruit regions, light-exposed (redder) and -unexposed (greener) peel regions were separated. For both blushed and striped fruit regions, peel tissue from at least two apples was pooled. In all cases, leaves and peels were immediately frozen in liquid nitrogen and placed at -80°C before anthocyanins, DNA or RNA was extracted.

### Identification and quantification of anthocyanins

Apple peel samples were finely ground in liquid nitrogen and then resuspended in 1 ml methanol and 0.1% HCl. Samples were sonicated for 4 min, stored at room temperature in the dark for 3 h and then centrifuged at 3,000 × g. Aliquots of 1.0 ml were dried down to completion in a Labconco Centrivap Concentrator (Labconco, Kansas City, MO, USA). Samples were resuspended in 20% methanol (250 μl). Anthocyanins were identified by LC-MS analysis as described previously [53]. Identification was based both on masses (M^+^) of molecular ions and characteristic fragments/neutral losses and comparison of retention times and fragmentation with authentic standards of cyanidin-3-O-glucoside and cyanidin-3-O-galactoside. M^+ ^fragments observed were 303 Da (delphinidin), 287 Da (cyanidin) and 271 Da (pelargonidin). Neutral losses (i.e. mass differences between fragments) observed were 162 Da (hexoside sugar, e.g. galactose), 146 Da (deoxyhexoside sugar, e.g. xylose) and 132 Da (pentoside sugar, e.g. arabinose). MS cannot distinguish between sugars of the same mass (e.g. glucose/galactose). Anthocyanins and other phenolic compounds were quantified by HPLC as described previously [53]. Quantification was achieved by reference to standards of anthocyanins and other phenolic compounds, using LC-MS data to confirm identification of peaks.

### Real-time transcript analysis

Mature 'Honeycrisp' fruit peels were very finely ground in liquid nitrogen and RNA was extracted using the Lopez-Gomez and Gomez-Lim extraction method [54] as modified by Mann et al. [55]. Briefly, after precipitation with 3 M LiCl, RNA was collected by centrifugation at 12,000 × g for 30 min, and second day LiCl washes were eliminated. RNA pellets were resuspended in 400 μl RNAse free sterile water, potassium acetate was added to a final concentration of 0.3 M, and the RNA was precipitated with two volumes of absolute ethanol. After incubation for at least 1 hour at 20°C, RNA was pelleted by centrifugation (20,000 × g for 30 min) and resuspended in RNAse free sterile water. RNA was treated with RQ1 RNAse-free DNAse (Promega Corp., Madison, WI) and then purified using the RNeasy RNA clean-up procedure (Qiagen, Valencia, CA). RNA quantification was performed using a Qubit™ fluorometer (Invitrogen Corp., Carlsbad, CA). Total RNA was reverse-transcribed into cDNA using the Super-Script III (Invitrogen Corp.) reverse transcriptase kit. Real-time PCR amplification and analysis were carried out using an Applied Biosystems 7500 real-time PCR system (Applied Biosystems, Foster City, CA). Reactions were performed in triplicate using 10 μl 2X iTaq SYBR Green Supermix with ROX (Bio-Rad, Hercules, CA) Master Mix, 1 μl 10 mM of each primer, 1 μl template and nuclease-free water (Qiagen) to a final volume of 20 μl. Primers were designed to amplify *actin CHS, CHI, F3H, DFR1, LDOX, UFGT, MYB10, MYB17, bHLH3 *and *bHLH33 *(Table 1). A negative nuclease-free water control was included in each run. PCRs used an initial denaturation step at 95°C for 3 min, followed by 40 cycles of denaturation for 15 s at 95°C and annealing and elongation for 60 s at 60°C. Fluorescence was measured at the end of each annealing step at 60°C. Amplification was followed by a melting curve evaluation. The data were analyzed with the Applied Biosystems Sequence Detection Software, version 1.4.0.25 (Applied Biosystems), and transcript levels were normalized to *Malus *× *domestica actin *(*MdActin*, Genbank accession number CN938023) to minimize variation in cDNA template levels. *Actin *was selected for normalization due to its consistent transcript levels throughout leaf and fruit tissues, with crossing threshold (Ct) values changing by less than 2. For each gene, a standard curve was generated using a cDNA serial dilution, and the resultant PCR efficiency calculations (ranging between 1.839 and 1.945) were used for relative transcript level analysis. Error bars shown in real-time PCR data are biological and technical replicates, representing the means ± SE of three biological samples and three technical replicates. Analysis of variance (ANOVA) on real-time PCR data was performed using JMP^® ^7.0 statistical software (SAS Institute Inc, Cary, NC). Student's t-test was used to establish significant differences in transcript accumulation between biological replicates.

**Table 1:** Forward and reverse primers used in real-time PCR and RT-PCR analyses

Gene identifier (Genbank)	Name	Forward primer	Reverse primer
CN938023	*Actin*	TGACCGAATGAGCAAGGAAATTACT	TACTCAGCTTTGGCAATCCACATC

CN944824	*CHS*	GGAGACAACTGGAGAAGGACTGGAA	CGACATTGATACTGGTGTCTTCA

CN946541	*CHI*	GGGATAACCTCGCGGCCAAA	GCATCCATGCCGGAAGCTACAA

CN491664	*F3H*	TGGAAGCTTGTGAGGACTGGGGT	CTCCTCCGATGGCAAATCAAAGA

AF117268	*DFR1*	GATAGGGTTTGAGTTCAAGTA	TCTCCTCAGCAGCCTCAGTTTTCT

AF117269	*LDOX*	CCAAGTGAAGCGGGTTGTGCT	CAAAGCAGGCGGACAGGAGTAGC

AF117267	*UFGT*	CCACCGCCCTTCCAAACACTCT	CACCCTTATGTTACGCGGCATGT

DQ267896	*MYB10*	TGCCTGGACTCGAGAGGAAGACA	CCTGTTTCCCAAAAGCCTGTGAA

CO867070	*MYB17*	TGGCTCCAGAAAAGCAAATCA	GGCCGCTTGCAGAATCTGTA

CN934367	*bHLH3*	AGGGTTCCAGAAGACCACGCCT	TTGGATGTGGAGTGCTCGGAGA

DQ266451	*bHLH33*	ATGTTTTTGCGACGGAGAGAGCA	TAGGCGAGTGAACACCATACATTAAAGG

DQ886414	*MYB10 *cDNA	GCGGTACCGGTAGCAGGCAAAAGAATAGCTAAGC	GCGGATCCCACATTTACAAGCAAGGAAAATA

AY603367	TRIM	CGGGATGTGACAATTTGGTA	GCGATGTGGGATGTTACAAT

EU518249	*MYB10 *-2029 to -1229	GAAATCGTTCGAAGGTCTAAGG	TTCGTTGGATTCCGTTAAGC

EU518249	*MYB10 *-1411 to -678	AACCTTCACAAGGGTTGTCG	AATGGATGGAATGGAACGAA

EU518249	*MYB10 *-677 to 47	TTCGTTCCATTCCATCCATT	AGTCCAGGCACCTTTTCTCA

EU518249	*MYB10 *-1007 to -684	TGGAGTTAAATTAAYAAGGY	ARARRARAAAATCCTARCCC

EU518249	*MYB10 *-534 to -184	GAATGAAGAAGAGGGAAAAAAA	ATCCACARAARCAAACACTRACA

Primers used to amplify anthocyanin biosynthetic enzymes, candidate transcription factors and TRIM transposable elements.

Mature 'Royal Gala' peel RNA was isolated by a method adapted from Chang et al. [56], quantified in a NanoDrop nd-100 spectrophotometer running software version 3.0.1 (NanoDrop Technologies, Wilmington, DE) and treated with DNA-free DNAse (Ambion, Austin, TX). For real time-PCR analysis, total RNA was reverse-transcribed into cDNA using the Super-Script III (Invitrogen Corp.) reverse transcriptase kit. Real-time PCR amplification and analysis were carried out using the Roche 480 LightCycler System (Roche Diagnostics, Mannheim, Germany). All reactions were performed using the LightCycler 480 SYBR Green I Master Mix (Roche Diagnostics) following manufacturer instructions. Reactions were performed in triplicate using 5 μl 5X Master Mix, 1.0 μM each primer and 3 μl diluted cDNA. A negative nuclease-free water (Roche Diagnostics) control was included in each run. Primers used are the same as described above. PCRs used an initial denaturation step at 95°C for 5 min, followed by 50 cycles of denaturation for 10 s at 95°C, annealing for 10 s at 60°C and elongation for 20 s at 72°C. Fluorescence was measured at the end of each annealing step at 72°C. Amplification was followed by a melting curve analysis with continual fluorescence data acquisition during the 65-95°C melt curve. The raw data were analyzed with the LightCycler software, (LightCycler 480, Software 1.5) and transcript levels were normalized to *actin *to minimize variation in cDNA template levels. For each gene, a standard curve was generated using a cDNA serial dilution, and the resultant PCR efficiency calculations (ranging between 1.81 and 2.0) were imported into relative transcript level analysis. Error bars shown in real-time PCR data are technical replicates, representing the means ± SE of three replicate real-time PCR reactions. ANOVA on real-time PCR data was performed as described above.

### *MYB10 *characterization

To study sequence diversity in the *MYB10 *coding region, fruit peel RNA and cDNA were obtained using the modified Lopez-Gomez and Gomez-Lim extraction method as described above. The *MYB10 *coding region was amplified using PfuUltra™ Hotstart DNA Polymerase (Stratagene, La Jolla, CA) using *MYB10 *cDNA primers (Table 1) and DNA template. Reactions were performed in a 50 μl total volume (15 ng template, 100 ng/μl each primer, 25 mM each dNTP, 2.5 units AmpliTaq™ (Applied Biosystems), 10X buffer provided by manufacturer and 25 mM MgCl_2_). PCRs used 35 cycles of 94°C 30 s, 50°C 30 s, 72°C 120 s (Gene Amp PCR system 9700, Applied Biosystems). Fragments were then A-tailed by incubating 3 μl PCR product with 1 μl AmpliTaq™ (Applied Biosystems), 1 μl buffer provided by manufacturer, 1 μl 2 mM dATP, and 1 μl sterile water for 24 minutes at 70°C. Fragments were then desalted through a MicroSpin™ S-200 HR column (Amersham Biosciences, Piscataway, NJ) according to manufacturer's recommendations. Desalted fragments were cloned into the pGEM^®^-T Easy Vector (Promega Corp.), also according to manufacturer's instructions. Plasmids were purified from 3 ml overnight cultures using the Wizard *Plus *SV Minipreps DNA Purification system (Promega Corp.). To verify insert size, 3 μl of plasmid DNA were digested in 1X manufacturer supplied buffer by 10 units *Eco*RI (Invitrogen) in a 10 μl total volume at 37°C for 1 h. The entire reaction was loaded and separated on 1% agarose gels in TBE buffer, stained with ethidium bromide, and photographed under UV light. Inserts were compared to DNA standards of known size. Subsequently, undigested plasmids were sequenced using 3.2 pM of M13 forward or reverse primer. All nucleotide sequences were determined by Applied Biosystems BigDye Terminator version 3.1 cycle sequencing on an Applied Biosystems 3130xl or 3730xl automatic sequencer (Applied Biosystems) at the University of Minnesota DNA Biomedical Genomics Center's sequencing and analysis facility. Sequences were analyzed, assembled into contigs, and compared to known sequences using SeqMan™ II (Windows 32 vs. 5.08; DNASTAR Inc, Madison, WI).

For characterization of the *MYB10 *region upstream of the translation start site (referred to as "promoter" for simplification), leaf tissues or fruit peels were very finely ground in liquid nitrogen, and DNA was isolated using the Haymes' method [57] or using the DNeasy Plant mini Kit (Qiagen). Three promoter regions were amplified using PfuUltra ™ Hotstart DNA Polymerase (Stratagene) using *MYB10 *primer pairs -2029/-1229, -1411/-678 and -677/47 (Table 1). Reactions were performed as described above, but without additional MgCl_2_. PCR fragments were then desalted through a MicroSpin™ S-300 HR column (Amersham Biosciences) according to manufacturer's recommendations and fragments from three independent replicate reactions per sample were sequenced directly using 3.2 pM of the corresponding forward and reverse primers, as detailed above.

To amplify the TRIM element in the cultivars studied, standard PCRs were performed using AmpliTaq™ (Applied Biosystems) in a 50 μl total volume (15 ng genomic DNA as template, 1 μM each TRIM primer (Table 1), 200 μM each dNTP, 1.25 units Taq, 10X buffer provided by manufacturer). PCRs used 35 cycles of 94°C 30 s, 55°C 30 s, 72°C 60 s. These same thermocycling conditions were used to study whether the TRIM element is transcribed in the cultivars studied. The template in transcription studies consisted of cDNA obtained as described above, and TRIM forward and reverse primers (Table 1) or TRIM forward combined with *MYB10 *-1873 were used (Table 2).

**Table 2:** Forward and reverse primers for apple genes used in McrBC/real-time PCR analyses

Primer position	Forward primer	Reverse primer
-2434 to -2281	TGTAACAAGATGATGACGACGTGTA	TCTCCGCTCCCCTTCCA

-2254 to -2098	CATTTCCACCGTTCATTTCTAAGTT	CAGTAGAGAGATGAAGAGGCAATGC

-2029 to -1873	GAAATCGTTCGAAGGTCTAAGG	ACAGCAAACACCCAAAATCC

-1874 to -1681	GTTGCCATTTTTGAACACAACA	CCACGTGTTCAGGGTCCTTT

-1708 to -1426	TTTAATAAAAAGGACCCTGAACACG	CGTGATATATGATCTTGATGGTTGA

-1411 to -1229	AACCTTCACAAGGGTTGTCG	TTCGTTGGATTCCGTTAAGC

-1094 to -891	GGTCCCGCAAGACAGATAACC	CACTAAAAAAACACTTAGGCATACGAA

-991 to -776	GGCTGAACCACCTATGAAAATAATG	AGACGCTACACCTAACACATTGCT

-846 to -651	CTCTTGTGAAAGCTTAGTGAGTTGAAG	TGAGAGGAATGGATGGAATGG

-704 to -555	CGGGCTAGGATTTTCTCCTCTT	CTTCTTCATTCCCCTCCTATTTGA

-543 to -450	GGAGAGAATCCTACTCCATAAATTACAAG	CTTTCGCTGCTTTTTCAAGTGTT

-465 to -316	GAAAAAGCAGCGAAAGCATGA	GGAAATCAATCCCAGGGCATA

-303 to -182	GTCGTGCAGAAATGTTAGCTTTTC	CAGAAGCAAACACTGACAAGTTTAAAAC

-168 to -45	TGCACGTCACTGGCCTTGTA	TAAGCTTAGCTATTCTTTTGCCTGCTA

-51 to 105	AGTGGGTAGCAGGCAAAAGA	TCCACTTTCCCTCTCCATGA

146 to 257	GAGCTGCAGACAAAGATGGTTAAA	CCTGTTTCCCAAAAGCCTGTGAAGT

494 to 710	ACCACAAACGTCGTCGTCAAC	CCAAAGGTCCGTGCTAAAGG

Primers used to amplify different regions of the *MYB10 *locus of apple (Genbank accession EU518249)

Primer position is indicated relative to the ATG translation start site.

### Methylation studies

Peel genomic DNA (less than 1 μg) from red or green stripes, or from red and green areas of blushed apples harvested in 2007 and 2008, was digested with McrBC (New England Biolabs, Beverly, MA) in 100 μl total volume including 1X NEB2 buffer, 0.1 mg/mL bovine serum albumin, 1 mM GTP and 40 U McrBC or 50% glycerol (mock digested reactions). Digestions were incubated overnight at 37°C to ensure complete digestion and then incubated at 65°C for 20 min to halt enzyme activity. Real-time experiments were performed on McrBC and mock digested template as described above for 'Honeycrisp' and primers used are presented in Table 2. For each experiment, real-time PCR runs, including a control (mock digested DNA) and a McrBC digested sample, were performed in triplicate, and two or three independent digestions were used. Percent methylation for individual samples was calculated as a function of the delta CT between control and McrBC treated DNA, using the formula:

Student's t-test was used to establish significant differences in template amounts between biological replicates and subsequently calculate sample size. The estimated sample size was used when determining whether significant differences occurred between red and green peel regions.

Peel genomic DNA from red or green stripes (2009 harvested apples) were subjected to bisulfite conversion using the EZ DNA Methylation-Gold™ Kit (Zymo Research Corp., Irvine, CA) following manufacturer's recommendations. Two *MYB10 *promoter regions were amplified using *MYB10 *primer pairs -1007/-684 and -534/-184 (Table 1). PCRs were performed using Zymo Taq™ DNA Polymerase (Zymo Research Corp.) in a 50 μl total volume (1.0 μl bisulfite converted DNA as template, 0.6 μM each primer, 250 μM each dNTP, 2.0 units Zymo Taq, 1X buffer provided by manufacturer). PCRs used an initial denaturation step at 95°C for 10 min, followed by of 35 cycles of 95°C 30 s, 50°C 40 s (-1007 to - 684 fragment) or 55°C (-534 to - 184 fragment), 72°C 60 s, and a final elongation step at 72°C for 7 minutes. A secondary PCR was carried out using the same primers and conditions, and 1.0 μl of the primary PCR product. Fragments were then desalted through a MicroSpin™ S-300 HR column (Amersham Biosciences) according to manufacturer's recommendations. Desalted fragments were cloned into the pGEM^®^-T Easy Vector (Promega Corp.), also according to manufacturer's instructions. Bacterial colonies were frozen in 100 μl aliquots of Luria broth (Miller) solution with 10% glycerol in 96-well plates and shipped on dry ice to Beckman-Coulter Genomic Services (Beverly, MA) for Sanger sequencing. Percent methylation was calculated based on the methylation status of each cytosine within the two regions sequenced, using 12 to 24 clones per sample.

## Authors' contributions

AT conceived of the study, participated in its design, carried out the molecular biology experiments and drafted the manuscript, JMB conceived of the study, participated in its design and coordination and helped draft the manuscript, ACA participated in the design and coordination of the study and helped draft the manuscript, KLW carried out DNA and RNA extractions and real-time experiments, DES carried out anthocyanin quantification analysis and edited the manuscript, JMC carried out identification of anthocyanin compounds using LC-MS, RPH participated in the design of the study and helped draft the manuscript and EEH participated in the design of the study and provided the majority of funding to complete the research. All authors read and approved the final manuscript.

## Supplementary Material

Additional file 1 **Overall methylation of individual clones in the *MYB10 *-1007 to -684 promoter region**. Percent methylation in the 48 clones obtained from red and green stripes is presented. Clones are sorted in ascending order according to methylation percentages. Regression lines for methylation levels in green and red stripes as a function of clone number are indicated as green and red dotted lines respectively, and highlight the higher values observed in green stripes as compared to red stripes. Click here for file

Additional file 2 **Sequence alignment of *MYB10 *and 48 individual clones in the -1007 to -684 promoter region**. Increased *MYB10 *DNA methylation in green stripes is evident when comparing the number of methylated cytosines in each nucleotide position (A). Yellow bands indicate the location of cytosines in *MYB10*. Bars indicate the net difference in methylation (expressed as number of clones) at a particular site. Green bars indicate that a larger number of green stripe-derived clones carry methylated cytosines in that particular nucleotide position; red bars indicate higher methylation in red stripe-derived clones. Panel B depicts a DNA sequence alignment of *MYB10 *clones obtained from bisulfite-treated DNA from green stripes (24 clones) and from red stripes (24 clones). Methylated cytosines are highlighted in yellow in all the sequences. Methylation, which is mostly present at the 5' and 3' ends of this region, was observed in all cytosine contexts. Click here for file
