# Supplementary figures and images for: Apple skin patterning is associated with differential expression of MYB10
Source: BMC Plant Biol. 2011 May 20;11:93. doi: 10.1186/1471-2229-11-93 (PMC3127826; doi:10.1186/1471-2229-11-93)

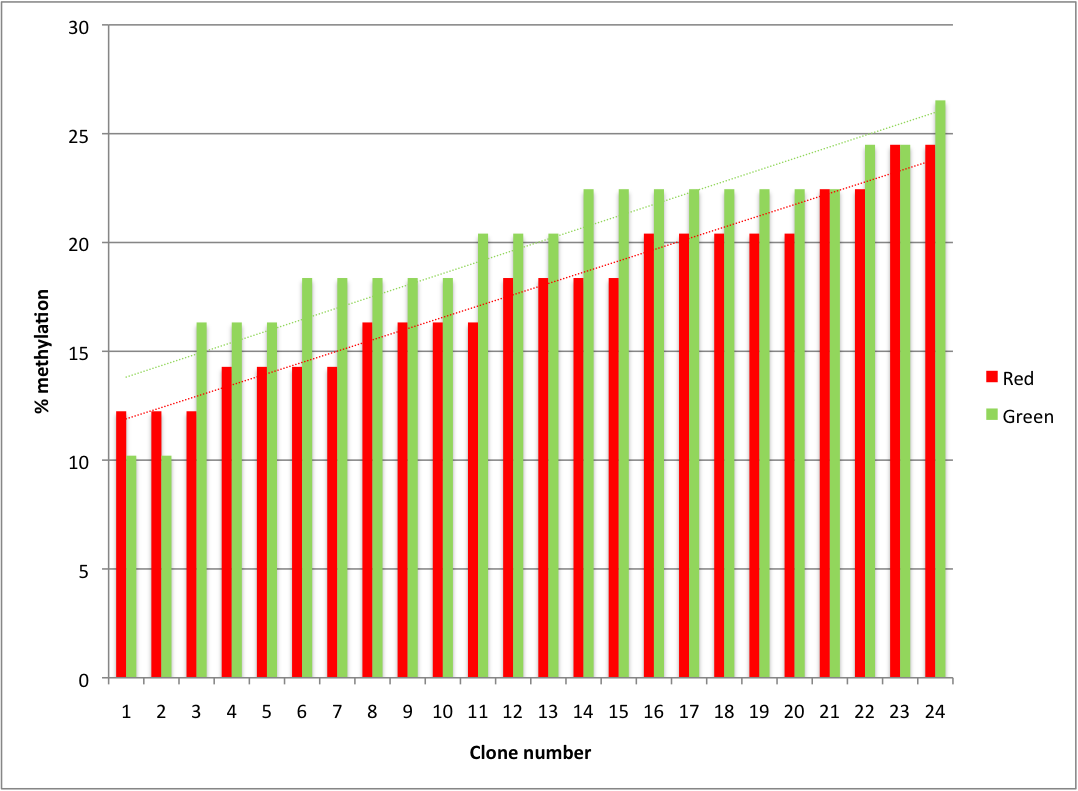

Supplement: Additional file 1 — Overall methylation of individual clones in the MYB10 -1007 to -684 promoter region. Percent methylation in the 48 clones obtained from red and green stripes is presented. Clones are sorted in ascending order according to methylation percentages. Regression lines for methylation levels in green and red stripes as a function of clone number are indicated as green and red dotted lines respectively, and highlight the higher values observed in green stripes as compared to red stripes. [file 1471-2229-11-93-S1.PNG]

## Slide 1
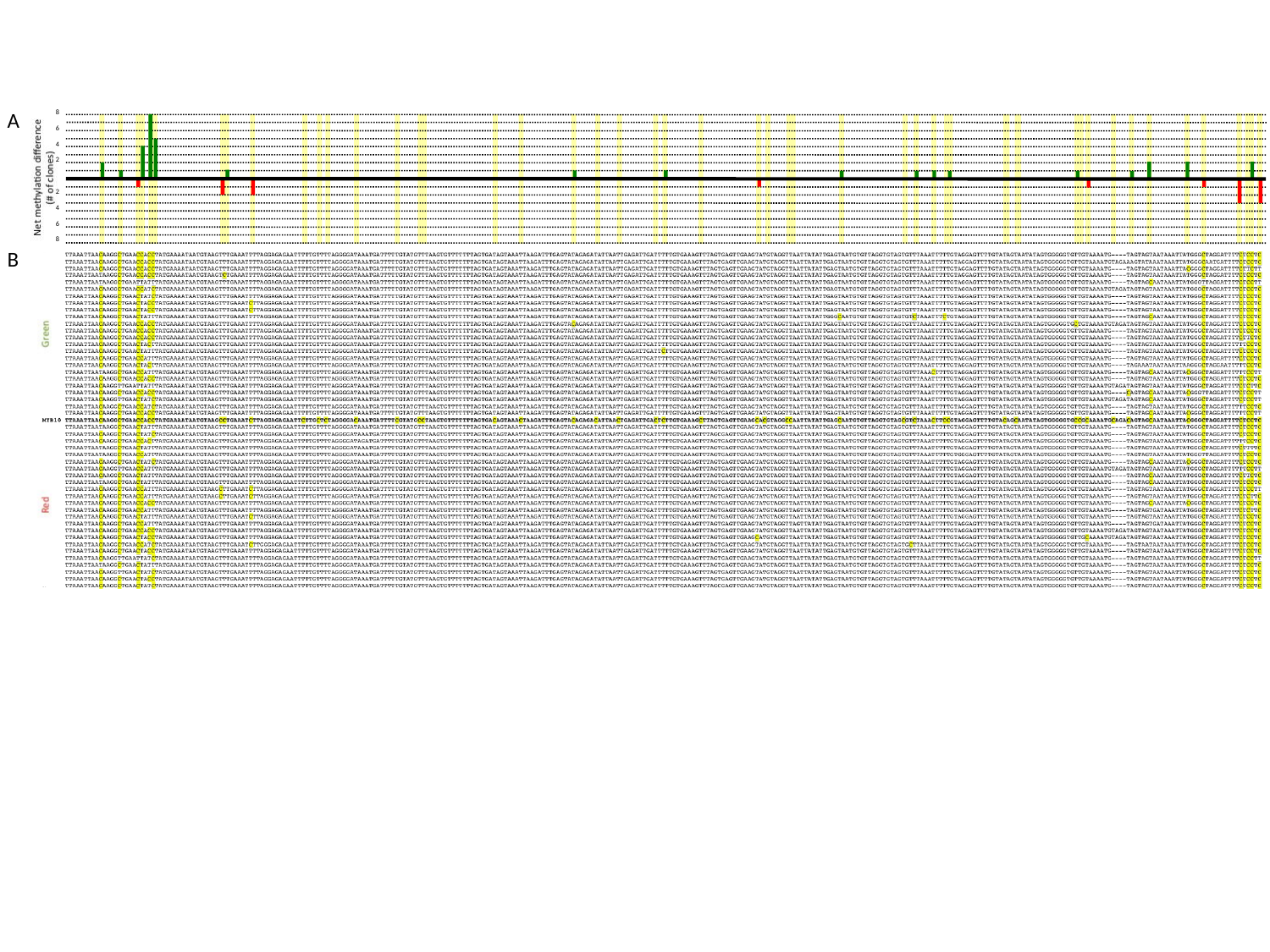

A
8
6
4
2
2
4
6
8
B

Supplement: Additional file 2 — Sequence alignment of MYB10 and 48 individual clones in the -1007 to -684 promoter region. Increased MYB10 DNA methylation in green stripes is evident when comparing the number of methylated cytosines in each nucleotide position (A). Yellow bands indicate the location of cytosines in MYB10. Bars indicate the net difference in methylation (expressed as number of clones) at a particular site. Green bars indicate that a larger number of green stripe-derived clones carry methylated cytosines in that particular nucleotide position; red bars indicate higher methylation in red stripe-derived clones. Panel B depicts a DNA sequence alignment of MYB10 clones obtained from bisulfite-treated DNA from green stripes (24 clones) and from red stripes (24 clones). Methylated cytosines are highlighted in yellow in all the sequences. Methylation, which is mostly present at the 5' and 3' ends of this region, was observed in all cytosine contexts. [file 1471-2229-11-93-S2.PPT]
